# Epigenetic inactivation of *mir-34b/c* in addition to *mir-34a* and *DAPK1* in chronic lymphocytic leukemia

**DOI:** 10.1186/1479-5876-12-52

**Published:** 2014-02-22

**Authors:** Lu Qian Wang, Yok Lam Kwong, Kit Fai Wong, Chi Shan Bonnie Kho, Dong Yan Jin, Eric Tse, Anders Rosèn, Chor Sang Chim

**Affiliations:** 1 Department of Medicine, Queen Mary Hospital, The University of Hong Kong, Hong Kong, China; 2 Department of Pathology, Queen Elizabeth Hospital, Hong Kong, China; 3 Department of Medicine, Pamela Youde Nethersole Hospital, Hong Kong, China; 4 Department of Biochemistry, The University of Hong Kong, Hong Kong, China; 5 Department of Clinical & Experimental Medicine, Linköping University, Linköping, Sweden

**Keywords:** MicroRNA, TP53 network, Tumor suppressor, DNA methylation, Chronic lymphocytic leukemia

## Abstract

**Background:**

*TP53* mutation/deletion is uncommon in chronic lymphocytic leukemia (CLL). We postulated that components of TP53-centered tumor suppressor network, *miR-34b/c*, in addition to *DAPK1* and *miR-34a* might be inactivated by DNA hypermethylation. Moreover, we tested if *miR-34b/c* methylation might correlate with *miR-203* or *miR-124-1* methylation in CLL.

**Methods:**

*miR-34b/c*, *miR-34a* and *DAPK1* methylation was studied in 11 normal controls, 7 CLL cell lines, and 78 diagnostic CLL samples by methylation-specific polymerase chain reaction. MEC-1 cells were treated with 5-Aza-2’-deoxycytidine for reversal of methylation-associated miRNA silencing. Tumor suppressor properties of *miR-34b* were demonstrated by over-expression of precursor *miR-34b* in MEC-1 cells.

**Results:**

*miR-34b/c* promoter was unmethylated in normal controls, but completely methylated in 4 CLL cell lines. *miR-34b/c* expression was inversely correlated with *miR-34b/c* methylation. Different MSP statuses of *miR-34b/c*, including complete methylation and complete unmethylation, were verified by quantitative bisulfite pyrosequencing. 5-Aza-2’-deoxycytidine treatment resulted in promoter demethylation and *miR-34b* re-expression in MEC1 cells. Moreover, over-expression of *miR-34b* resulted in inhibition of cellular proliferation and increased cell death. In primary CLL samples, *miR-34a*, *miR-34b/c* and *DAPK1* methylation was detected in 2.6%, 17.9% and 34.6% of patients at diagnosis respectively. Furthermore, 39.7%, 3.8% and 2.6% patients had methylation of one, two or all three genes respectively. Overall, 46.2% patients had methylation of at least one of these three genes. Besides, *miR-34b/c* methylation was associated with methylation of *miR-34a* (P = 0.03) and *miR-203* (P = 0.012) in CLL.

**Conclusions:**

Taken together, *miR-34b/c* is a tumor suppressor miRNA frequently methylated, and hence silenced in CLL. Together with *DAPK1* methylation, *miR-34b/c* methylation is implicated in the disruption of the TP53-centered tumor suppressor network. Moreover, the association of miRNA methylation warrants further study.

## Background

DNA methylation refers to the chemical modification of the cytosine ring in a CpG dinucleotide by the addition of a methyl group (-CH_3_) to the 5-carbon position of the cytosine pyrimidine ring in DNA, leading to the formation of 5-methylcytosine (5meC) [1]. Global DNA hypomethylation and aberrant, locus-specific DNA methylation of the promoter-associated CpG islands of tumor suppressor genes (TSGs) are the hallmarks of many human cancers [2-4]. It has been reported that methylation of TSG results in dysregulation of the cell cycle (*CDKN2A*/*B*), apoptosis (*DAPK1*/*CDKN2A*/*APAF1*), WNT (soluble WNT inhibitors) and JAK/STAT signaling (SOCS1 and PTPN6) in leukemia, lymphoma and myeloma, suggesting that TSG methylation plays an important role in the pathogenesis of various hematological cancers [5-8]. In chronic lymphocytic leukemia (CLL), TSGs including *DAPK1, SFRP1* or *SFRP2* have been shown to be aberrantly methylated (hypermethylated) [9-11].

Mature microRNA (miRNAs) are a class of endogenous, single-stranded, non-protein-coding small RNAs measuring 19 to 25 nucleotides (nts), which are responsible for the downregulation of targeted protein [12,13]. miRNAs involved in carcinogenesis may be either oncogenic (oncomirs) or tumor suppressive [14]. Recently, tumor suppressive miRNAs have also shown to be silenced by aberrant DNA methylation in cancers [1].

Recently, DNA methylation of *miR-34b/c* has been demonstrated in colorectal cancer, lung cancer and acute lymphoid leukemia. *miR-34b/c*, like *miR-34a*, is under the transcriptional regulation of *TP53. TP53* inactivation, mostly via mutations, occurs in up to 50% epithelial cancers at diagnosis [15,16]. In contrast, *TP53* mutation or del (17p) is only found in only 5% to 10% of CLL patients at diagnosis [17-19]. Therefore, other mechanisms leading to *TP53* malfunction may exist. In this connection, methylation of *DAPK1* gene, which triggers *TP53* activation upon oncogenic cellular transformation, was first reported as a TSG methylated in CLL [20], and subsequently shown to contribute to CLL progression by blocking the apoptosis of leukemia cells [10]. Moreover, since the *miR-34* family is a transcriptional target of TP53, its methylation might lead to the perturbation of the TP53 tumor suppression pathway. In this report, we performed a comprehensive methylation study of the TP53-centered tumor suppressor network by studying DNA methylation of *miR-34a* and *DAPK1* in addition to *miR-34b/c* in a representative cohort of CLL.

## Materials and methods

### Patient samples

This study has been approved by the Institutional Review Board of Queen Mary Hospital and written informed consent has been obtained in accordance with the Declaration of Helsinki. Diagnostic bone marrow samples were obtained in 78 CLL patients. Diagnosis of CLL was made according to the WHO Classification, which is based on classical morphology, low level of expression of light-chain-restricted surface immunoglobulin, and dual positivity of CD5 and CD23 in the neoplastic lymphocytes as demonstrated by flow cytometry [21,22]. There were 51 (65.4%) male and 27 female (34.6%) patients with a median age of 65 years (range: 37–91 years). The median presenting lymphocyte count was 18 × 10^9^/l (range: 10 – 540 × 10^9^/l). Among 70 patients with information for Rai staging, there were 42 (60.0%) patients with limited Rai stage (< stage II) and 28 (40.0%) with advanced Rai stage (≥ stage II) disease. Of the 48 patients with cytogenetic data, 12 (25.0%) carried high/intermediate-risk cytogenetic aberrations [del (17p), N = 2; del (11q), N = 1; trisomy 12, N = 9] and 36 (75.0%) carried low/standard-risk cytogenetic alterations [del (13q), N = 11; normal karyotype, N = 18; other karyotypic changes, N = 7]. The median overall survival (OS) of the cohort was 69 months. The median OS of those with Rai stage > 2 and those Rai stage ≤ 2 were 49 and 111 months respectively (P = 0.006). Moreover, the median OS for those with or without high/intermediate-risk karyotype were 28 months and 111 months (P = 0.003). Of these, the *miR-34a* and *DAPK1* methylation results of 50 patients have previously been reported [20,23]. Therefore, in this study, *DAPK1* and *miR-34a* methylation were studied in an additional 28 diagnostic CLL primary samples.

### Cell lines and culture

The CLL cell lines MEC1 and CLL-AAT were purchased from Deutsche Sammlung von Mikroorganismen und Zellkulturen Deutsche GmbH (DMSZ) (Braunschweig, Germany) and American Type Culture Collection (Manassas, USA) respectively. MEC2, WAC3CD5+ and I83-E95 were kindly provided by Dr John C. Byrd, Department of Medicine, Ohio State University [24,25]. Moreover, HG3 and 232B4 were kind gifts from Prof. Anders Rosén, Department of Clinical & Experimental Medicine, Linköping University [25,26]. Cell lines were maintained in 90% RPMI 1640 + 10% FBS. Culture media were supplemented with 50 U/ml penicillin and 50 μg/ml streptomycin (Invitrogen, Carlsbad, CA, USA), and maintained in a humidified atmosphere of 5% CO_2_ at 37°C.

### Methylation-specific polymerase chain reaction (MSP)

DNA was extracted from bone marrow samples of CLL at diagnosis, the cell lines and 11 normal controls (peripheral blood buffy coats obtained from 5 healthy donors, bone marrow buffy coat from 3 healthy donors, and CD19 sorted peripheral blood B-cells from 3 healthy controls) by standard method. The normal controls comprised 5 males and 6 females with a median age of 38 years (range: 25–60 years). Treatment of DNA with bisulfite for conversion of unmethylated cytosine to uracil (but unaffecting methylated cytosine) was performed with a commercially available kit (EpiTect Bisulfite Kit, QIAGEN, Hilden, Germany). Details of primers and conditions for MSP of *miR-34b/c*, *miR-34a* and *DAPK1* were given in Table 1. To define the sensitivity of *miR-34a* M-MSP, 1 μg of methylated control DNA was 10-foldedly serially diluted in buffer, bisulfite-treated and amplified with *miR-34a* M-MSP primers.

**Table 1:** ***miR-34a*, *miR-34b/c *** **and *DAPK1 *** **MSP Primer sequences and the reaction condition**

**Gene**	**Forward primer (5’ to 3’)**	**Reverse primer (5’ to 3’)**	**Tm/cycles/MgCl**_**2**_	**References**
***miR-34a***				
U-MSP	GGGGATGAGGATTAGGATTTT	CAAACAAAACACATAAAAACAACA	58°C/35×/1.5 mM	[23]
M-MSP	GGGGATGAGGATTAGGATTTC	ACAAAACGCATAAAAACGACG	58°C/35×/1.5 mM	
***miR-34b/c***				
U-MSP	TTTTTATTTGTTTTGTTTTGTGTTTGTTTTG	CAACTACAACTCCCAAACAATCC	56.5°C/38×/2 mM	[27,28]
M-MSP	ATTCGTTTCGTTTCGCGTTCGTTTC	CGACTACAACTCCCGAACGATCCG	60°C/34×/2 mM	
***DAPK1***				
U-MSP	GGAGGATAGTTGGATTGAGTTAATGTT	CAAATCCCTCCCAAACACCAA	63°C/35×/1.5 mM	[20]
M-MSP	GGATAGTCGGATCGAGTTAACGTC	CCCTCCCAAACGCCGA	63°C/35×/1.5 mM	

*Abbreviations*: *M-MSP* MSP for the methylated allele, *U-MSP* MSP for the unmethylated allele, *Tm* annealing temperature.

### Quantitative bisulfite pyrosequencing

DNA was treated with bisulfite and used as template. Primers for pyrosequencing were used to amplify the promoter region, which was overlapped with the amplicon of MSP. Primers were designed using PSQ Assay Design software (Biostage). Forward primer: 5’- GGAAGGGGAGGTTTGGTA-3’; Reverse primer: 5’-ACCACCACAATACAATCAACTAATA-3’; condition: 2 mM/59°C/50X. A stretch of DNA with 12 adjacent CpG dinucleotides was pyrosequenced by sequencing primer: 5’-CAACTAATAACACTACCTACA-3’.

### 5-Aza-2’-deoxycytidine (5-AzadC) treatment

MEC1 cells were cultured in six-well plates at 1 × 10^6^ cells/ml, with 0.5 μM of 5-AzadC (Sigma-Aldrich, St. Louis, MO, USA) for 5 days. Cells on day 0 and day 5 of 5-AzadC treatment were harvested.

### Quantification of *miR-34b* and reverse transcription-PCR of *DAPK1*

According to the respective manufacturer’s instructions, total RNA was isolated and reversely transcribed using the mirVana miRNA Isolation Kit. miRNA was quantified by the TaqMan MicroRNA RT Kit, and TaqMan MicroRNA Assay Kit as reported [27-29]. RNU48 was chosen as reference for data analysis using the 2^-∆∆^Ct method [30]. Moreover, for semi-quantitative analysis of *DAPK1* expression, *DAPK1* was reversely transcribed by the QuantiTect Reverse Transcription Kit (QIAGEN, Valencia, CA) [31]. Reverse transcription-PCR primers and PCR condition for *DAPK1* were summarized in Table 2.

**Table 2:** **Primer sequences and the reaction condition of *DAPK1 *** **and *GAPDH *** **Reverse transcription-polymerase chain reaction (RT-PCR)**

**Gene**	**Forward primer (5’ to 3’)**	**Reverse primer (5’ to 3’)**	**Tm/cycles/MgCl**_**2**_	**References**
*DAPK1*	CAGTTTGCGGTTGTGAAGAA	CCTGCAACGAGTTCCAAGAT	53°C/35×/2 mM	[31]
*GAPDH*	ACCACAGTCCATGCCATCACT	TCCACCACCCTGTTGCTGTA	60°C/24×/2 mM	[23]

*Abbreviations*: *Tm* annealing temperature.

### Western blot for DAPK1

MEC1, MEC2, 232B4, CLL-AAT and WAC3CD5+ cells were harvested and then lysed in RIPA buffer (50 mM Tris–HCl, pH 7.4, 150 mM NaCl, 0.2% SDS, 1% Triton X-100, 2 mM EDTA). Protein lysates were resolved on 6% SDS-PAGE and electrotransferred onto a 0.2 μm nitrocellulose membrane (Bio-Rad, Hercules, CA). The membranes were blocked and incubated with anti-DAPK1 (1:1000; Sigma-Aldrich, USA) or anti-actin (1:5000; Sigma-Aldrich, USA) primary antibody at 4°C overnight. Then membranes were washed three times and incubated with anti-rabbit horseradish peroxidase conjugate secondary antibody at room temperature for 1 hour. Protein signals were detected by ECL Prime Western blotting detection reagents (Amersham Biosciences, Buckinghamshire, UK).

### Precursor *miR-34b* overexpression

Precursor *miR-34b* (100nM; Ambion) (oligonucleotide mimic) was transfected into 1 × 10^6^ MEC1 cells using X-tremeGENE siRNA Transfection Reagent (Roche Diagnostics/Roche, Basel, Switzerland), according to the manufacturers’ instructions [28]. Non-targeting oligonucleotide mimic was used as negative control.

### Proliferation, viability and cell cycle analyses

To document the tumor suppressor function of *miR-34b*, three independent transfections were performed, in which functional studies including MTT assay, Trypan blue exclusion assay and the percentage of sub-G1 fraction were performed in triplicate after each transfection. The MTT method was used to determine cellular proliferation. Cells were cultured in a 96-well microtitre plate at 2.5 × 10^4^/well in 100 μl of medium. At test time-points, 10 μl of 5 mg/ml MTT reagent was added to each well and incubated for 4 hours, after which 100 μl of dimethyl sulfoxide (DMSO) was added, and absorbance at 550 nm with reference to 650 nm was measured. Cellularity viability assay was performed by the Trypan blue dye exclusion assay. For cell cycle analysis, test cells were washed in phosphate buffered saline (PBS), fixed in cold 70% ethanol at 4°C overnight, washed twice in PBS, resuspended and incubated in 50 μg/ml PI staining solution with 5 μg/ml RNase A at 4°C for at least 2 hours, and then analyzed by flow cytometry (Beckman Coulter Cytomics FC 500).

### Statistical analysis

In CLL, correlation between *DAPK1, miR-34a* and *miR-34b/c* methylation status with continuous (mean age, mean diagnostic haemoglobin (Hb), lymphocyte or platelet counts at diagnosis) and categorical variables (gender, Rai stage or high-risk karyotypes) were studied respectively by Student’s t-test and chi-square test (or Fisher’s exact test). Moreover, in 50 samples, the methylation of *miR-203*, *miR-124-1* has been studied [29,32], and the association of *miR-34b/c* with the methylation of *miR-203* and *miR-124-1* was analyzed by chi-square test. OS is measured from the date of diagnosis to the date of last follow-up or death. OS of patients with limited Rai stage (stages 0, I and II) was compared to those with advanced Rai stage (stages III and IV). Moreover, OS of patients with high-risk karyotypes [del (17p), del (11q) or trisomy 12] was compared with those with standard-risk karyotypes [del (13q), normal karyotype or other karyotypic changes]. The mean values of MTT assay, Trypan blue exclusion assay and sub-G1 fraction in MEC1 cells transfected with precursor *miR-34b* mimic were compared with negative control transfected with a scrambled oligo by Student’s t-test. Survival is plotted by the Kaplan–Meier method and compared by the log-rank test. All P values were two-sided.

## Results

### MSP

#### ***Controls***

**Figure 1:**
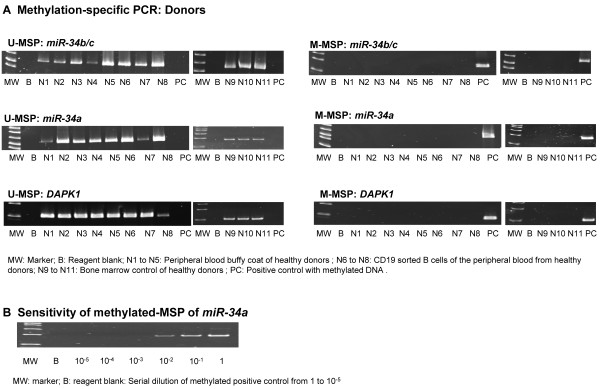
**MSP of *miR-34b/c*, *miR-34a *and *DAPK1 *in controls. (A)** U- and M-MSP of *miR-34b/c, miR-34a* and *DAPK1* showed that the positive control (PC) was completely methylated while all normal controls were completely unmethylated. MW: Marker; B: Reagent blank; N1 to N5: Peripheral blood buffy coat of healthy donors; N6 to N8: CD19 sorted B cells of the peripheral blood from healthy donors; N9 to N11: Bone marrow control of healthy donors; PC: Positive control with methylated DNA. **(B)** Sensitivity of methylated-MSP of *miR-34a.* To define the sensitivity of *miR-34a* M-MSP, 1 μg of methylated control DNA was 10-fold serially diluted in buffer, bisulfite-treated and amplified with *miR-34a* M-MSP primers*.* MW: marker; B: reagent blank: Serial dilution of methylated positive control from 1 to 10^-5^.

None of the 8 normal peripheral blood controls (N1 to N8) and 3 normal bone marrow controls (N9 to N11) showed aberrant methylation of *miR-34b/c*, *miR-34a* or *DAPK1* (Figure 1A). Expected MSP results (normal DNA: U-MSP positive/M-MSP negative; methylated DNA: U-MSP negative/M-MSP positive) were demonstrated in the positive and negative controls. Moreover, the sensitivity of *miR-34a* M-MSP was 10^-2^ (Figure 1B).

#### ***CLL cell lines***

**Figure 2:**
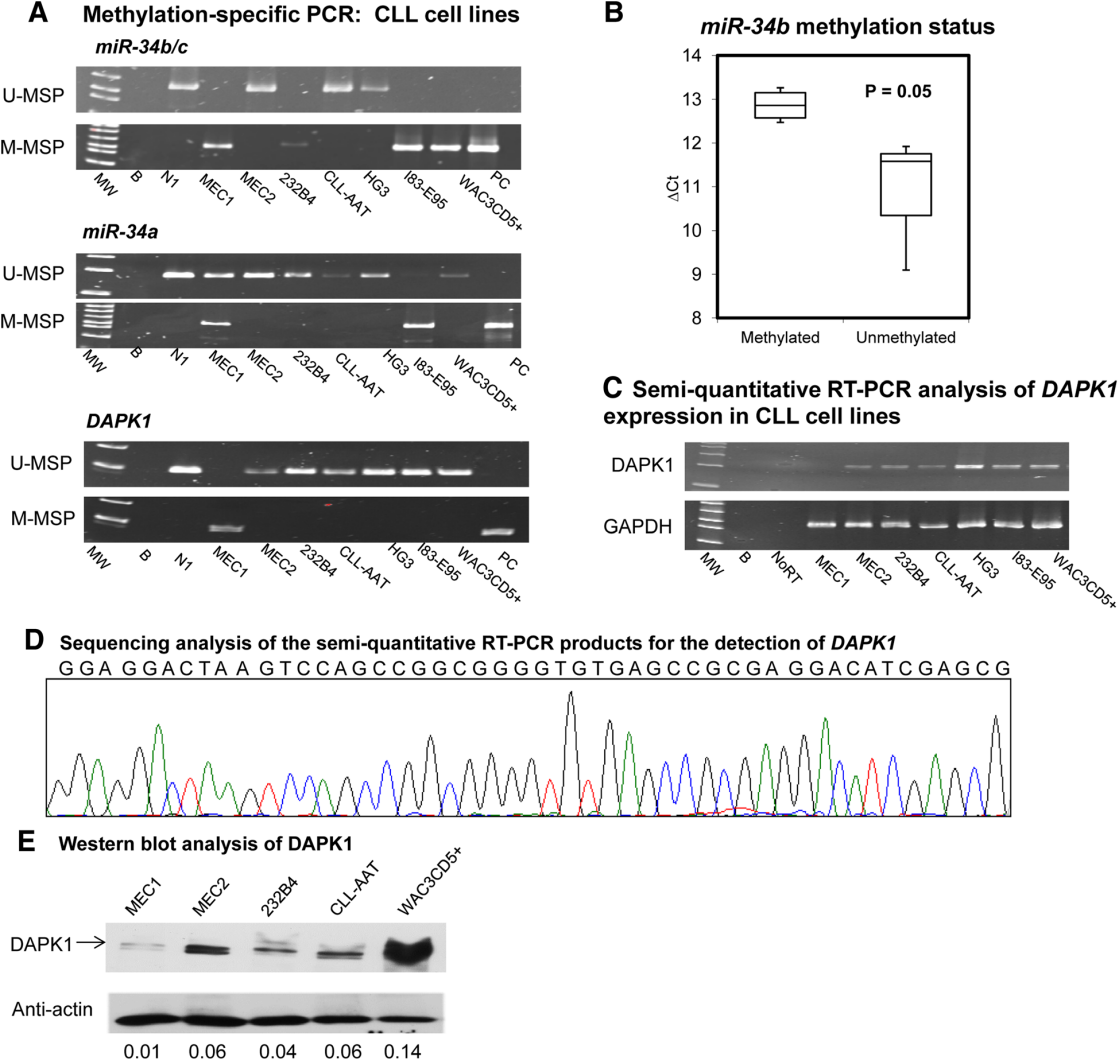
**Methylation of *miR-34b/c*, *miR-34a *and *DAPK1 *in CLL cell lines. (A)** In CLL cell lines, MEC1, 232B4, I83-E95 and WAC3CD5+ were completely methylated and MEC2, HG3 and CLL-AAT were completely unmethylated for *miR-34b/c*. I83-E95 was completely methylated, MEC1 was partially methylated, but MEC2, 232B4, CLL-AAT, HG3 and WAC3CD5+ were unmethylated for *miR-34a*. Moreover, MEC1 was completely methylated, and other six cell lines were unmethylated for *DAPK1*. **(B)** Stem-loop qRT-PCR analysis of the mature *miR-34b* expression in 7 CLL cell lines. ∆Ct, Ct *miR-34b*-Ct RNU48. **(C)** In CLL cell lines, MEC1 cells showed no *DAPK1* mRNA expression while other six cell lines had detectable *DAPK1* mRNA levels. **(D)** Sequencing analysis of the semi-quantitative RT-PCR products for the detection of *DAPK1.* MW: Marker; B: Reagent blank; N1: Normal donors; NoRT: Negative control without reverse transcriptase; PC: positive control with methylated DNA. **(E)** Western blot analysis of DAPK1 in MEC1, MEC2, 232B4, CLL-AAT and WAC3CD5+ cells. WAC3CD5+ cells were used as positive control. Anti-actin protein was regarded as the endogenous normalizer and the relative DAPK1 protein level was shown in the bottom row.

The profile of *miR-34b/c* methylation of 7 CLL cell lines was shown in Figure 2A. MEC1, 232B4, I83-E95 and WAC3CD5+ showed complete methylation of *miR-34b/c* whereas MEC2, HG3 and CLL-AAT were completely unmethylated. Quantitative bisulfite pyrosequencing confirmed the methylation statuses (MM and UU) of CLL cell lines detected by MSP (Additional file 1: Table S1 and Figure S1A-C). On the other hand, *miR-34a* was completely methylated in I83-E95, partially methylated in MEC1 and completely unmethylated in MEC2, 232B4, CLL-AAT, HG3 and WAC3CD5+ (Figure 2A). *DAPK1* was completely methylated in MEC1 but completely unmethylated in other six CLL cell lines (Figure 2A).

Moreover, in order to confirm the association of *miR-34b/c* expression and methylation in CLL cell lines, the mean *miR-34b* expression and *miR-34b/c* methylation level were compared among the 7 CLL cell lines. The results showed the mean *miR-34b* expression in completely methylated CLL cell lines (MEC1, 232B4, I83-E95 and WAC3CD5+) was significantly lower than that of unmethylated MEC2, HG3 and CLL-AAT, and hence a higher (∆Ct) (P = 0.05) (Figure 2B). Furthermore, in CLL cell lines, complete methylation of *DAPK1* in MEC1 cells showed absence of *DAPK1* mRNA expression while other six cell lines without *DAPK1* methylation showed detectable *DAPK1* mRNA levels (Figure 2C). The sequence analysis of semi-quantitative RT-PCR of *DAPK1* was shown in Figure 2D. Western blot analysis also revealed marked downregulation of DAPK1 protein expression in MEC1 when compared with cells with absence of *DAPK1* methylation including MEC2, 232B4, CLL-AAT, WAC3CD5+ cells (Figure 2E).

### Primary samples at diagnosis

#### ***(I) miR-34 family***

**Figure 3:**
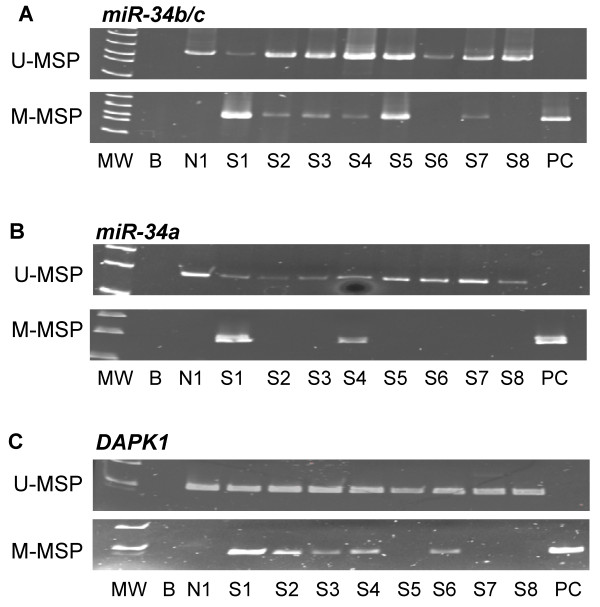
**Promoter methylation of *miR-34b/c*, *miR-34a *and *DAPK1 *in CLL primary samples. (A)** U-/M-MSP analysis of *miR-34b/c* methylation in CLL primary samples. **(B)** U-/M-MSP analysis of *miR-34a* methylation in CLL primary samples. **(C)** U-/M-MSP analysis of *DAPK1* methylation in CLL primary samples. MW: Marker; B: Reagent blank; N1: Normal donors; S: Sample at diagnosis; PC: Positive control with methylated DNA.

*miR-34b/c* methylation was found in 14 of 78 (17.9%) patient samples at diagnosis (Figure 3A). No correlation was demonstrated between *miR-34b/c* methylation and the diagnostic Hb level (P = 0.76), lymphocyte count (P = 0.51) or platelet count (P = 0.65). There was no significant association of *miR-34b/c* methylation with age (P = 0.97), gender (P = 0.76), advanced Rai stage (≥ stage 2) (P = 0.75) and high-risk karyotypic aberrations (P = 0.66). The median OS of CLL patients with and without *miR-34b/c* methylation were 51 and 69 months respectively (P = 0.77). In addition, *miR-34a* methylation was present only in 2 (2.6%) of CLL samples (Figure 3B).

Moreover, methylation status of *miR-203, miR-34a* and *miR-124-1* was available in 50 patients. *miR-34b/c* methylation was found to be associated with the methylation of *miR-203* (P = 0.012) and *miR-34a* (P = 0.03), but not *miR-124-1* (P = 0.06).

#### ***(II) DAPK1***

*DAPK1* was found in 27 of 78 (34.6%) patients at diagnosis (Figure 3C). Apart from the association with advanced age (P = 0.04), the methylation status of *DAPK1* was not associated with other clinical demographics including gender (P = 0.46) and advanced Rai stage (≥ stage 2) (P = 0.30), high-risk karyotypic aberrations (P = 0.73), diagnostic Hb level (P = 0.67), lymphocyte count (P = 0.51) or platelet count (P = 0.59). The median OS for CLL patients with and without *DAPK1* methylation were 89 and 68.88 months respectively (P = 0.98). The methylation status of *DAPK1* was not associated with that of *miR-34b/c* (P = 0.99), *miR-34a* (P = 0.12), *miR-124a* (P = 0.40) or *miR-203* (P = 0.13).

**Figure 4:**
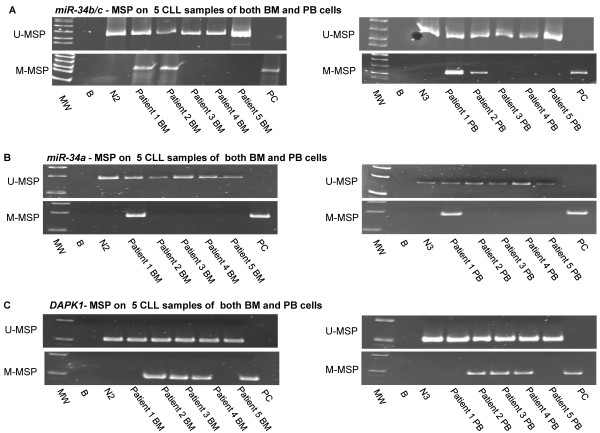
**Comparison of the MSP results of *miR-34b/c*, *miR-34a *and *DAPK1 *between the bone marrow cells and peripheral blood cells of the same CLL patients.** U-/M-MSP analysis of **(A)***miR-34b/c*, **(B)***miR-34a* and **(C)***DAPK1* on 5 CLL primary samples of both bone marrow and peripheral blood cells. MW: Marker; B: Reagent blank; N2: Normal bone marrow control; N3: Normal peripheral blood control; Patient BM: Bone marrow cells of CLL sample at diagnosis; Patient PB: Peripheral blood cells of CLL sample at diagnosis; PC: Positive control with methylated DNA.

Moreover, we performed MSP of *miR-34b/c, miR-34a* and *DAPK1* in both peripheral blood and bone marrow samples of patients, in whom both peripheral blood and bone marrow were available. Concordant MSP results of *miR-34b/c, miR-34a* and *DAPK1* between bone marrow cells and peripheral blood cells were demonstrated, and hence both peripheral blood and marrow tumor cells are valid for methylation study (Figure 4A-C).

### Methylation profiling of *miR-34a*, *miR-34b/c* or *DAPK1* in primary samples

Among the 78 patients, apart from 53.8% (42/78) of patients who did not show methylation of any of these three genes, 39.7% (31/78), 3.8% (3/78) and 2.6% (2/78) of patients had methylation of one, two or all of the three genes respectively. Overall, 46.2% (36/78) had methylation of at least one of these three genes.

### 5-AzadC treatment of MEC1 cells

**Figure 5:**
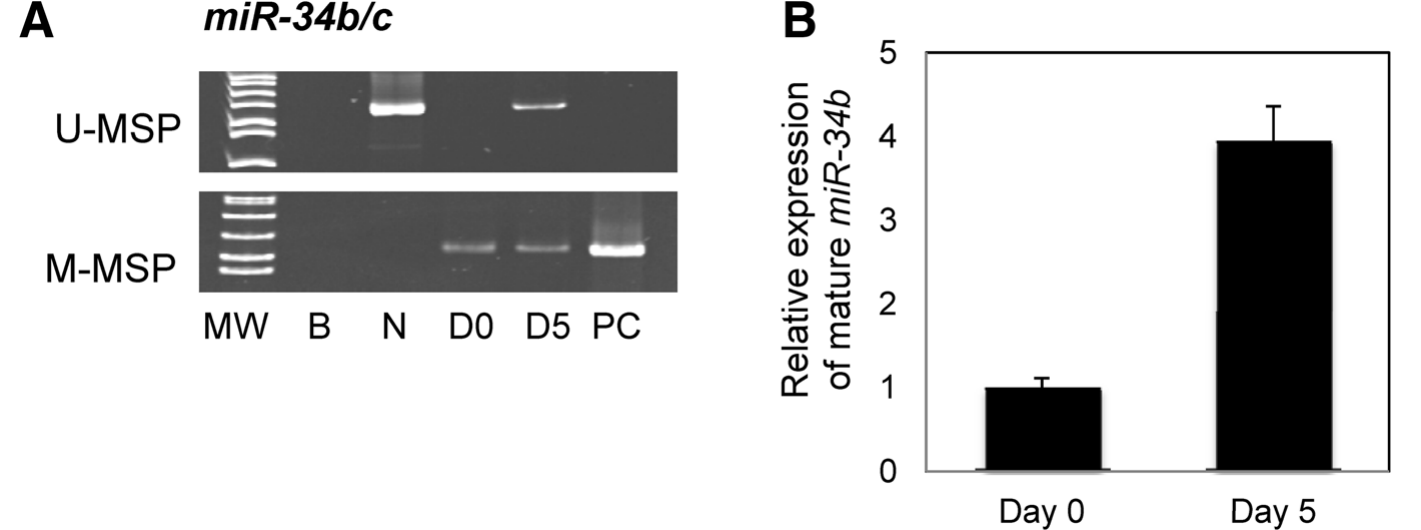
**Effect of 5-Aza-2’-deoxycytidine (5-AzadC) treatment on MEC1 cells. (A)** U- /M-MSP analysis of *miR-34b/c* promoter methylation status. **(B)** stem-loop RT-qPCR analysis of the mature *miR-34b* expression. 5-AzadC treatment led to the progressive demethylation of *miR-34b/c* promoter, and re-expression of the mature *miR-34b* in MEC1 cells. MW: Marker; B: Reagent blank; N: Normal donors; PC: Positive control with methylated DNA; D0: Day 0; D5: Day 5 culture in 0.5 μm 5-AzadC.

MEC1 cells were completely methylated for *miR-34b/c*. 5-AzadC demethylation treatment of MEC1 cells led to the demethylation of *miR-34b/c* and the emergence of U-MSP signal on day 5 (Figure 5A and Additional file 1: Figure S1D), with the re-expression of mature *miR-34b* shown by TaqMan stem-loop quantitative RT-PCR (Figure 5B).

### Effect of *miR-34b* overexpression in MEC1 cells

**Figure 6:**
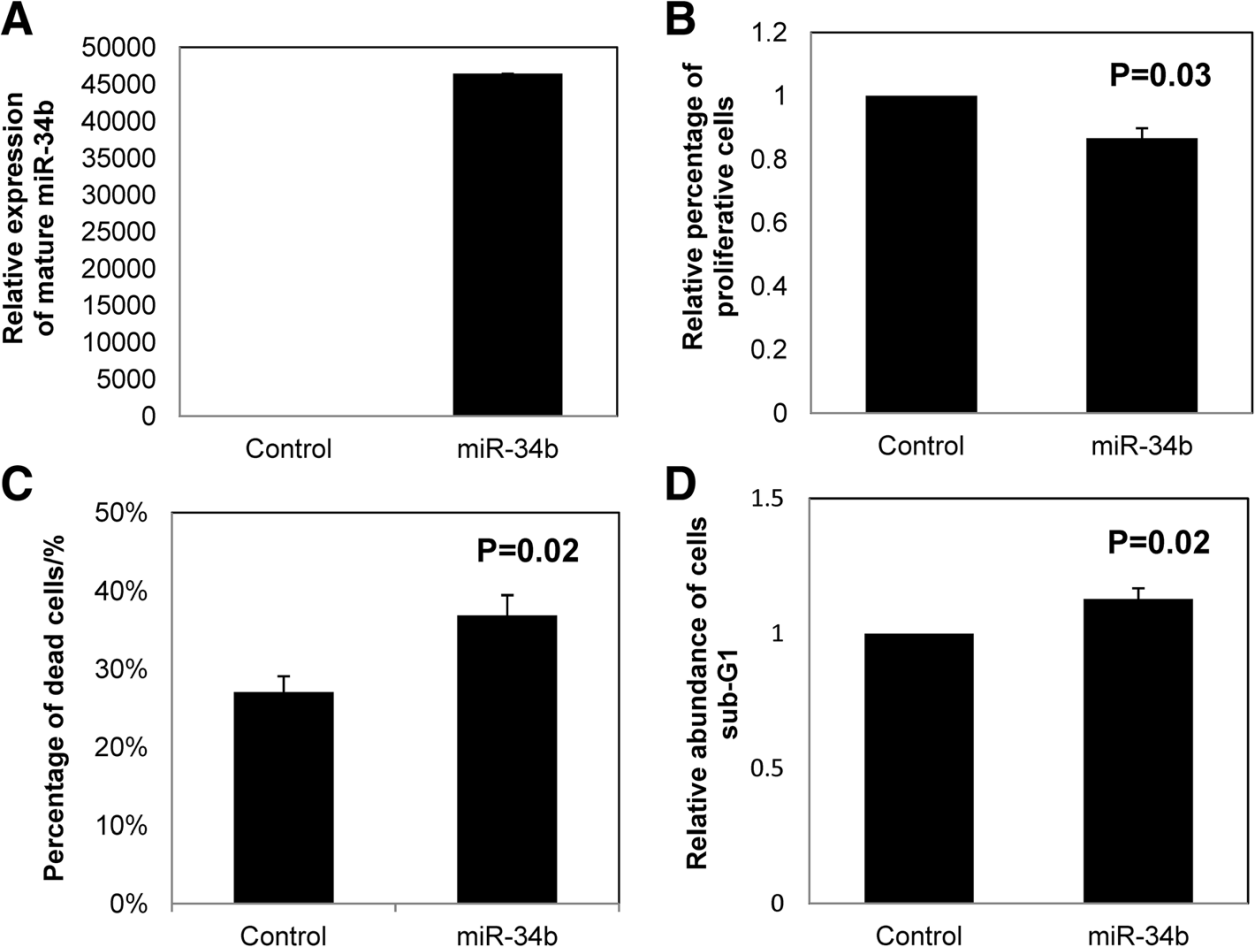
**Over-expression of *miR-34b *in CLL cells.** MEC1 cells, completely methylated for *miR-34b*, were transfected with mature *miR-34b* mimic or scrambled control oligos. **(A)** Stem-loop RT-qPCR analysis of mature *miR-34b* expression at 48 hrs after transfection. **(B)** Cell proliferation of CLL cells in response to overexpression of *miR-34b* was measured by MTT assay. **(C)** Cellular death was measured by Trypan blue exclusion assay. **(D)** The percentage of apoptotic cells (sub-G1 cells) was measured by the Propidium iodide staining. Values represented the average of the means from each of the 3 experiments performed in triplicate ± standard deviation.

*miR-34b* was completely methylated for the MEC1 cells, and hence under-expressed. Upon transfection of precursor *miR-34b* mimic into MEC1 cells, overexpression of mature *miR-34b* was demonstrated by TaqMan stem-loop quantitative RT-PCR (Figure 6A). When compared with negative control transfected with a scrambled oligo, cells over-expressing *miR-34b* mimic showed a 13% reduction of cellular proliferation by MTT assay (P = 0.03, Figure 6B), a 10% increase of dead cells measured by Trypan blue exclusion assay (P = 0.02, Figure 6C), in addition to a 12% increase of cells in sub-G1 phase using propidium iodide staining (P = 0.02, Figure 6D), suggesting that *miR-34b* played a tumor suppressive role in CLL cells.

## Discussion

Despite the retrospective nature, our cohort was representative of CLL in view of the prolonged survival, and the expected adverse impact of advanced Rai stage and high-risk karyotype. It is ideal to perform pyrosequencing of primary samples. Unfortunately, only buffy coat has been collected from the primary marrow samples without cell selection. Given that the sensitivity of M-MSP of *miR-34b/c* and *DAPK1* is 10^-3^[7,28], and that the leukemia infiltration ranged from 36% to 95% (median: 64%), our results are still valid even without CD19 cell sorting of primary samples. Moreover, in patients in whom both peripheral blood and bone marrow buffy coats were available, concordant MSP results for *miR-34b/c, miR-34a* and *DAPK1* were demonstrated, and hence both peripheral blood and bone marrow were valid for methylation analysis. Based on these experiments, several observations were made from the study.

Firstly, *miR-34b/c* methylation is tumor-specific as illustrated by the absence of methylation in normal peripheral blood and bone marrow cells, but complete methylation in 4 of the 7 CLL cell lines. Moreover, the expression of *miR-34b* was also correlated inversely with the methylation status, with low miRNA expression in completely methylated cell lines and significantly higher expression in completely unmethylated cell lines. Furthermore, after the 5-AzadC treatment of MEC1 cells, the appearance of U-MSP signal, and hence demethylation of *miR-34b/c* promoter, was associated with mature *miR-34b* re-expression. Therefore, hypermethylation of *miR-34b/c* is prevalent in CLL cell lines, resulting in reversible miRNA silencing.

Secondly, *miR-34b/c* is a tumor suppressor miRNA in CLL. This is demonstrated by the inhibition of cell proliferation and increase of cellular death upon over-expression of *miR-34b* in MEC1 cells possessing complete methylation of *miR-34b/c.* Given the increasing evidence that TSGs are silenced by gene hypermethylation, promoter hypermethylation, together with inactivating gene mutation or deletion of the other allele may serve as one of the two hits of Knudson’s hypothesis [2,33]. Moreover, *miR-34b/c* is localized to 11q23 and hence, it is tempting to postulate that *miR-34b/c* methylation might cooperate with del (11q) to inactivate both alleles of *miR-34b/c*, thereby fulfilling the Knudson’s hypothesis [34]. However, in our samples, there was only one case of del (11q) to verify this hypothesis.

In contrast to *miR-34a*, which was rarely hypermethylated in CLL, *miR-34b/c* was frequently methylated in CLL samples at diagnosis. However, there was no significant association between the methylation status of *miR-34b/c* with clinical parameters, such as age, gender, diagnostic Hb, lymphocyte or platelet count, Rai stage, or survival. However, it has been reported that the down regulation of *miR-34* family is involved CLL with an aggressive course [35]. In view of the small number of samples in our cohort, the impact of *miR-34b/c* methylation on survival warrants a larger scale study. Nevertheless, infrequent *miR-34a* methylation was also contrasted with the frequent *miR-34a* methylation in many epithelial cancers [23,36].

Frequent methylation of *DAPK1* was also observed in our patients, and 46.2% (36/78) had methylation of either *DAPK1* or *miR-34b/c*. Therefore, despite infrequent *TP53* deletion or mutation in CLL at diagnosis, frequent *miR-34b/c* or *DAPK1* methylation is implicated in the disruption of the TP53-centered tumor suppression network. This is particularly important as TP53 is also found to be haploinsufficient in carcinogenesis as inactivation of only one TP53 allele is sufficient to predispose to carcinogenesis [37].

Finally, an interesting observation was that methylation of *miR-34b/c* (localized at chromosome 11q23) was associated with methylation of *miR-203* (localized at 14q32), similar to the association between *miR-203* methylation with *miR-34a, miR-124a, miR-196b* and *miR-129-2* methylation in non-Hodgkin’s lymphoma [29,38]. *miR-34b/c* and *miR-203* have been shown independently to target *CREB* mRNAs [39,40]. As a transcription factor, CREB can upregulate the expression of multiple genes involved in the cell cycle progression (*CCNA1*, *CCNB1* and *CCND1*) and survival (*BCL2* and *NFκB1*) [39]. Therefore, concomitant methylation of both *miR-203* and *miR-34b/c* might collaborate in the de-repression of CREB-related cell proliferation and survival, contributing to carcinogenesis.

## Conclusion

In conclusion, *miR-34b/c* methylation is a tumor suppressor miRNA frequently methylated in CLL. *miR-34b/c* methylation is associated with reversible miRNA silencing. Despite the infrequent occurrence of inactivating *TP53* mutation in CLL at diagnosis, *miR-34b/c* methylation, together with *DAPK1* methylation, is implicated in the perturbation of the TP53-centered tumor suppressor network. Finally, the implication of association of *miR-34b/c* methylation with that of *miR-203* warrants further analysis.

## Abbreviations

CLL: Chronic lymphocytic leukemia; TSGs: Tumor suppressor genes; miRNAs: microRNAs; MSP: Methylation-specific polymerase chain reaction; 5-AzadC: 5-Aza-2’-deoxycytidine.

## Competing interests

The authors confirm that there are no conflicts of interest.

## Authors’ contributions

Conceived and designed the experiments: CSC and YLK. Acquisition of data: CSC, KFW, CSBK and ET. Analyzed the data: CSC, LQW and DYJ. Performed the experiments: LQW. Writing, review, and/or revision of the manuscript: LQW, CSC, YLK, KFW, CSBK, DYJ, ET and AR. All authors read and approved the final manuscript.

## Supplementary Material

Additional file 1: Table S1 Average percent methylation for *miR-34b/c* in 7 CLL cell lines by pyrosequencing. **Figure S1.** Quantitative bisulfite pyrosequencing analysis of *miR-34b/c*. The pyrograms showed the methylation intensity on a stretch of 12 neighboring CpG dinucleotides of (A) Normal control without methylation and positive control with methylated DNA, (B-C) CLL cell lines with defined MSP methylation status (MM and UU) and (D) MEC1 cells before and after 5-azadC treatment. Click here for file
