# Supplementary material for: Epigenetic inactivation of mir-34b/c in addition to mir-34a and DAPK1 in chronic lymphocytic leukemia
Source: J Transl Med. 2014 Feb 22;12:52. doi: 10.1186/1479-5876-12-52 (PMC3941938; doi:10.1186/1479-5876-12-52)

Table S1 Average percent methylation for *miR-34b/c* in 7 CLL cell lines by pyrosequencing

CLL cell Lines (n=7)	Ave.% Methylation (12 CpGs) by pyrosequencing	Methylation Status by MSP
I83-E95	86.33%	MM
WAC3CD5+	82.67%	MM
MEC1	81.00%	MM
232B4	35.33%	MM
CLL-AAT	22.91%	UU
MEC2	10.58%	UU
HG3	8.00%	UU

Table S1. **Quantitative bisulfite pyrosequencing analysis of *miR-34b/c*.** The table showed the average percent methylation for *miR-34b/c* (12 CpG sites) in 7 CLL cell lines, which were defined MSP methylation status (MM and UU). Primers for pyrosequencing were used to amplify the promoter region of *miR-34b/c*, which was overlapped with the amplicon of MSP.

Figure S1A

Normal control
Expected: 0%
Observed: 4.83%

Positive control
Expected: 100%
Observed: 81.75%

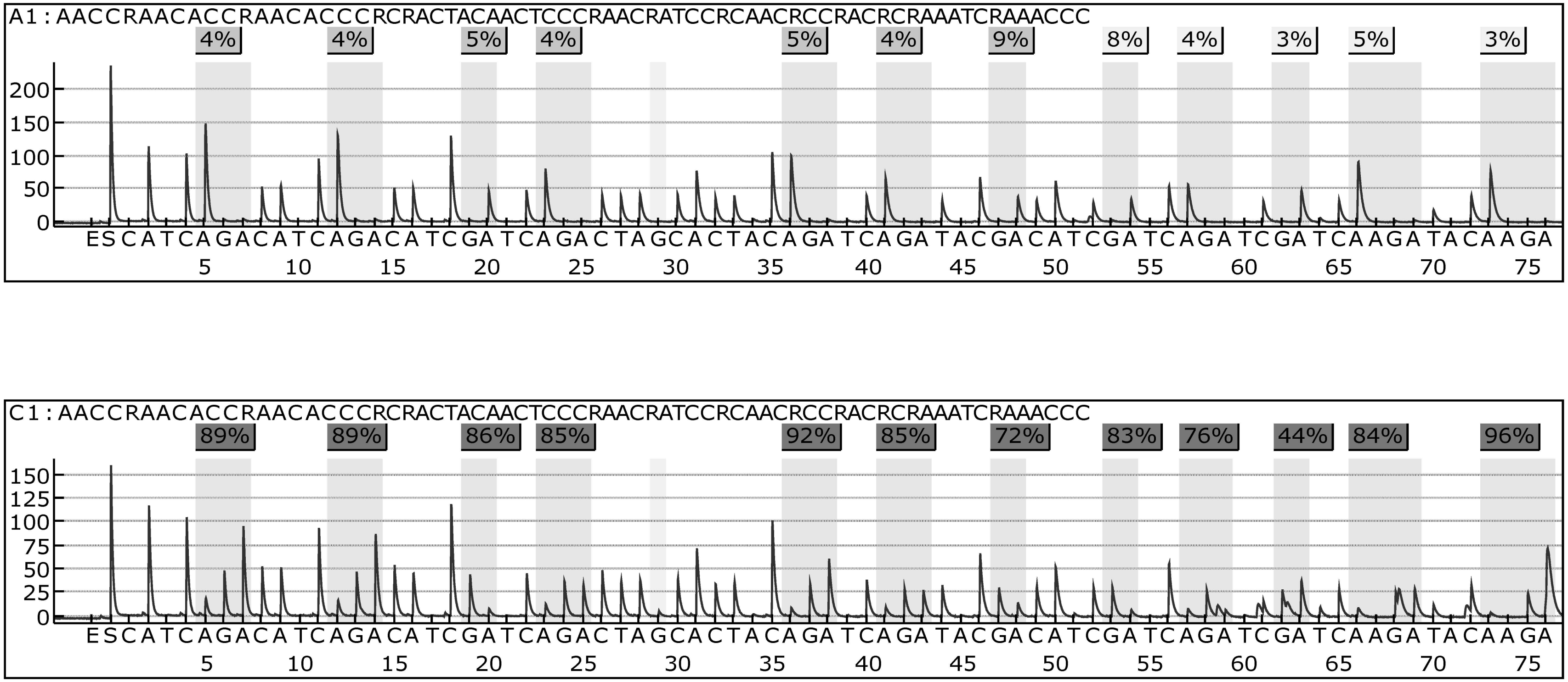

Figure S1. Quantitative bisulfite pyrosequencing analysis of *miR-34b/c*. The pyrograms showed the methylation intensity on a stretch of 12 neighboring CpG dinucleotides of (**A**) Normal control without methylation and positive control with methylated DNA, **(B-C)** CLL cell lines with defined MSP methylation status (MM and UU) and **(D)** MEC1 cells before and after 5-azadC treatment.

Figure S1B

I83-E95
MSP: MM
Pyro: 86.33%

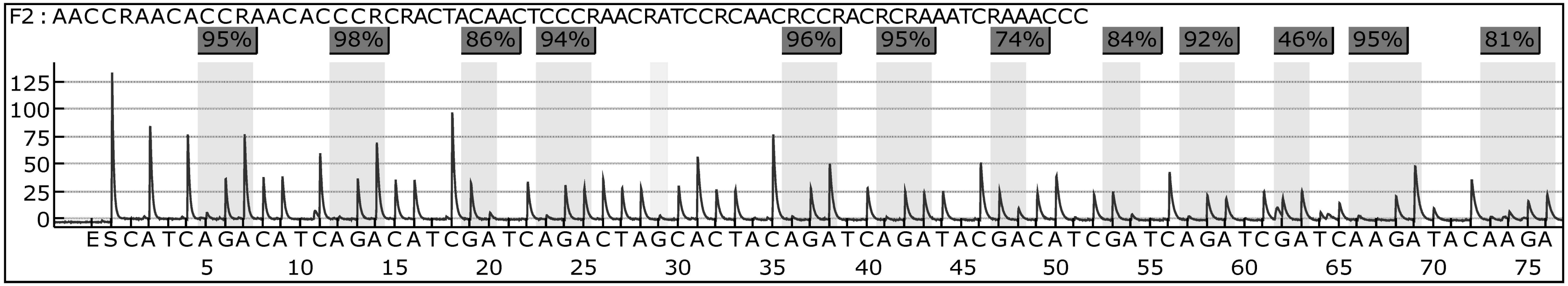

WAC3CD5+
MSP: MM
Pyro: 82.67%

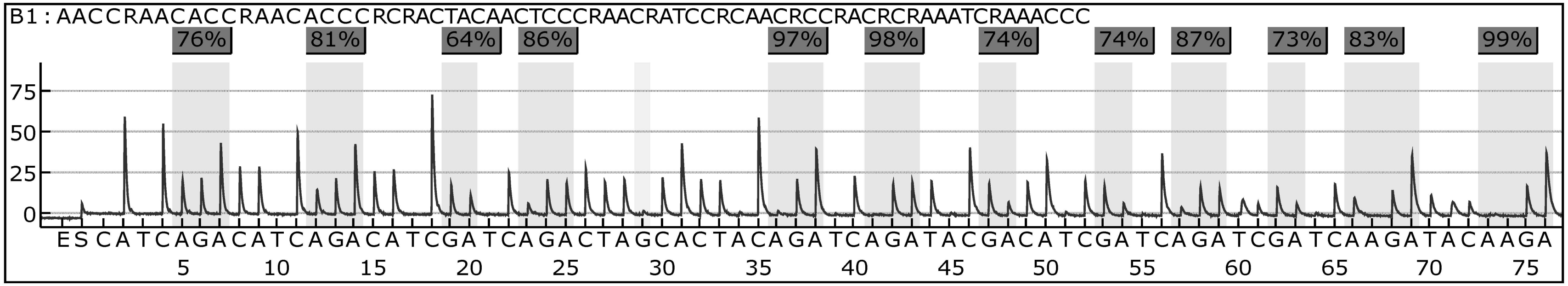

232B4
MSP: MM
Pyro: 35.33%

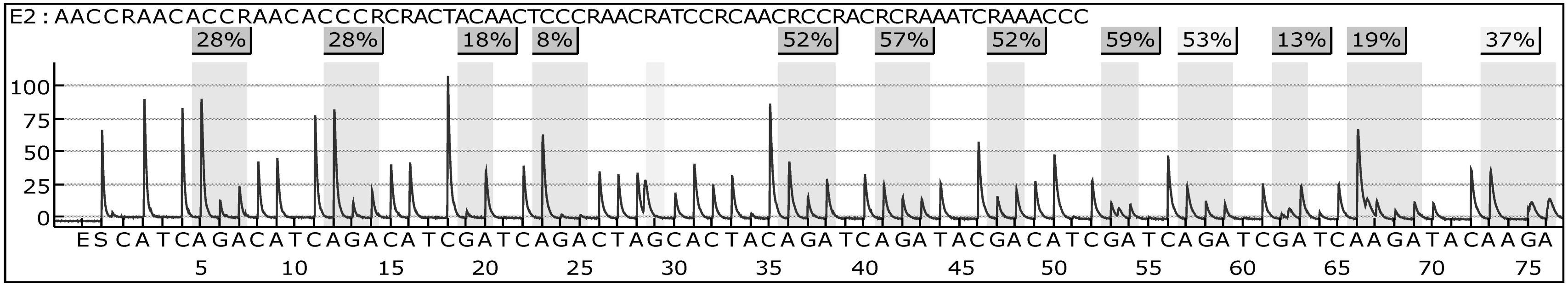

Figure S1C

HG3
MSP: UU
Pyro: 8.00%

MEC2
MSP: UU
Pyro: 10.58%

CLL-AAT
MSP: UU
Pyro: 22.91%

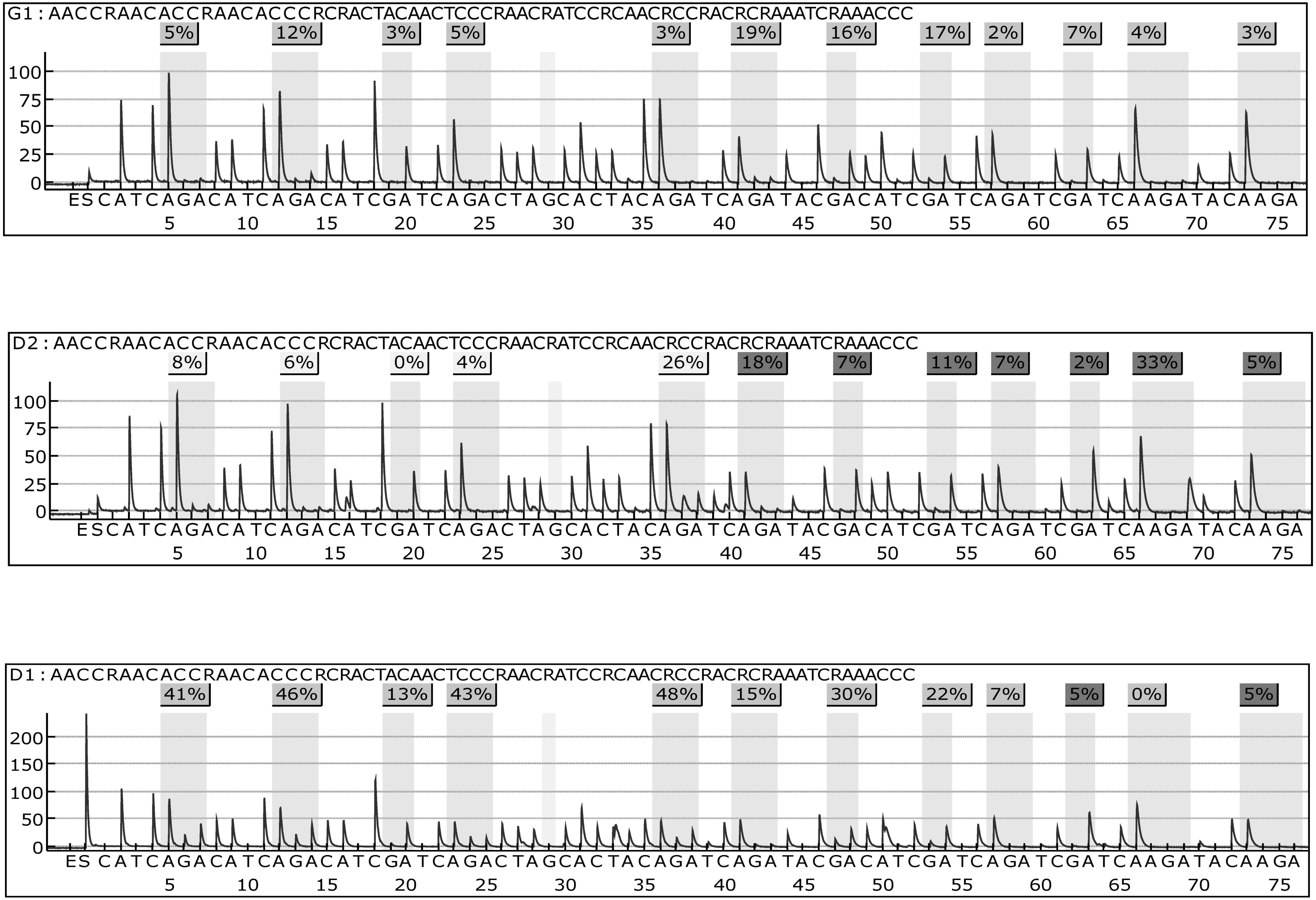

Figure S1D

MEC1
5-azadC: Untreated
MSP: MM
Pyro:81.00%

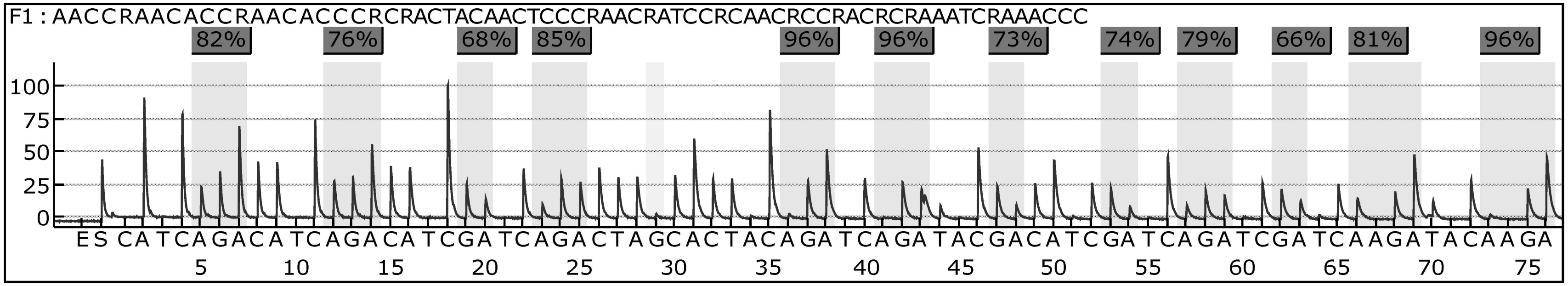

MEC1
5-azadC: 5 Day, 0.5μM
MSP: MU
Pyro:53.58%

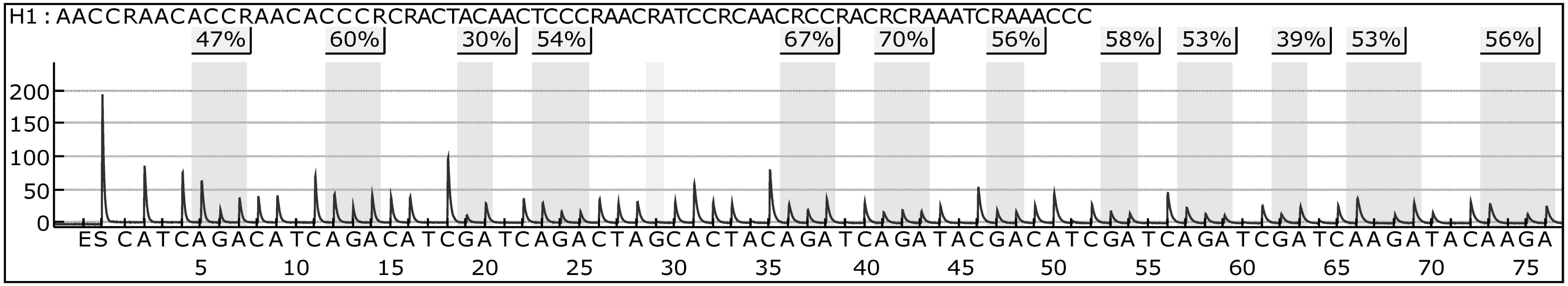

Supplement: Additional file 1: Table S1 — Average percent methylation for miR-34b/c in 7 CLL cell lines by pyrosequencing. Figure S1. Quantitative bisulfite pyrosequencing analysis of miR-34b/c. The pyrograms showed the methylation intensity on a stretch of 12 neighboring CpG dinucleotides of (A) Normal control without methylation and positive control with methylated DNA, (B-C) CLL cell lines with defined MSP methylation status (MM and UU) and (D) MEC1 cells before and after 5-azadC treatment. [file 1479-5876-12-52-S1.pdf]
